# General Sensitization of Melanoma Cells for TRAIL-Induced Apoptosis by the Potassium Channel Inhibitor TRAM-34 Depends on Release of SMAC

**DOI:** 10.1371/journal.pone.0039290

**Published:** 2012-06-18

**Authors:** Sandra-Annika Quast, Anja Berger, Nicole Buttstädt, Kristin Friebel, Roland Schönherr, Jürgen Eberle

**Affiliations:** 1 Department of Dermatology and Allergy, Skin Cancer Center, University Medical Center Charité, Berlin, Germany; 2 Institute for Chemistry and Biochemistry, Free University of Berlin, Berlin, Germany; 3 Department of Biophysics, Center of Molecular Biomedicine, Jena University Hospital, Jena, Germany; University of South Florida College of Medicine, United States of America

## Abstract

The death ligand TRAIL represents a promising therapeutic strategy for metastatic melanoma, however prevalent and inducible resistance limit its applicability. A new approach is presented here for sensitization to TRAIL. It is based on inhibition of the membrane potassium channel KCa3.1 (IK1), which serves fundamental cellular functions related to membrane potential. The selective inhibitor TRAM-34 did not induce apoptosis by itself but synergistically enhanced TRAIL sensitivity and overrode TRAIL resistance in a large panel of melanoma cell lines. Expression of IK1 was also found in mitochondria, and its inhibition resulted in mitochondrial membrane hyperpolarization and an early activation of Bax. The combination of TRAM-34 and TRAIL resulted in massive release of mitochondrial factors, cytochrome c, AIF and SMAC/DIABLO. Bax knockdown and Bcl-2 overexpression abolished apoptosis. Overexpression of XIAP diminished apoptosis by two-fold, and SMAC knockdown almost completely abolished apoptosis. These data uncover the existence of a rheostat in melanoma cells, consisting of inhibitor of apoptosis proteins and SMAC, which regulates TRAIL sensitivity. Thus, a new strategy is described based on mitochondrial membrane channels, which correspond to Bax activation. As both TRAIL and IK1 inhibitors had shown only minor side effects in clinical trials, a clinical application of this combination is conceivable.

## Introduction

Metastatic melanoma is characterized by unbroken high mortality [1]. The pronounced resistance to chemotherapy and to an anti-tumor immune response is related to defects in proapoptotic signaling [2]. Overcoming apoptosis resistance thus appears as a promising therapeutic goal. The death ligand TRAIL (TNF-related apoptosis-inducing ligand) induces apoptosis via TRAIL-R1/DR4 and TRAIL-R2/DR5 [3], whereas decoy receptors TRAIL-R3/DcR1 and TRAIL-R4/DcR2 lack the death domain and may block the pathway [4], [5]. TRAIL reveals the particular advantage of selectively targeting cancer cells, while normal cells are largely protected [6].

We have previously shown activity of DR4 and DR5 in melanoma cells. As the receptors were also expressed in melanoma tissue, TRAIL-based strategies appear as promising also for melanoma therapy [7]. Permanent TRAIL resistance, seen in some melanoma cell lines, as well as inducible resistance, seen in initially sensitive cell lines, may however limit its applicability [7], [8]. Induced TRAIL resistance had been correlated in melanoma cells with downregulation of TRAIL receptors, initiator caspases and proapoptotic Bcl-2 proteins [8], [9].

Two main branches of extrinsic and intrinsic apoptosis pathways have been described [10]. Extrinsic pathways are initiated by binding of TNF-α, CD95L/FasL or TRAIL to death receptors, formation of death-inducing signaling complexes (DISC) and activation of initiator caspases-8 and -10 [11]. On the other hand, intrinsic pathways are initiated by cellular and DNA damage and particularly employ mitochondria. The mitochondrial level is critically controlled by the family of pro- and antiapoptotic Bcl-2 proteins [12]. Key events are depolarization of the mitochondrial membrane potential (Δψm) and mitochondrial outer membrane permeabilization (MOMP), resulting in release of mitochondrial factors such as cytochrome c, AIF (apoptosis-inducing factor) and SMAC (second mitochondria-derived activator of caspases) [13]. Whereas cytochrome c results in activation of initiator caspase 9 [14], apoptosis by AIF was reported as caspase-independent [15].

The initiator caspases -8, -9 and -10 activate downstream effector caspases -3, -6 and -7, which cleave a large number of death substrates to set apoptosis into work [16]. Effector caspases and caspase-9 are critically inhibited by cIAPs (inhibitor of apoptosis proteins), which thus can prevent extrinsic and intrinsic pathways. Particularly, XIAP (chromosome x-linked IAP) has been attributed a decisive role in apoptosis resistance of tumor cells [17]. IAPs themselves are negatively regulated by SMAC, which is released from mitochondria upon apoptotic stimulation and binds to IAPs in a competitive manner, thus releasing caspase activity [18].

Membrane ion channels serve fundamental cellular functions. The group of Ca^2+^-dependent potassium channels contributes to cytoplasma membrane hyperpolarization thus facilitating Ca^2+^ entry, a prerequisite for cell proliferation [19]. The family member KCa3.1 (IK1) is inhibited by clotrimazole, commonly used in the clinic as fungicide, as well as by the scorpion venom charybdotoxin. Systemic application of clotrimazole is however prevented because of hepatotoxicity resulting from non-specific effects on cytochrome P450. The alternative analogue TRAM-34 lacks P450-inhibitory activity, thus avoiding these side effects [20].

Expression of IK1 was related to aberrant cell proliferation of different types of tumor cells [19], [21]. Induction of apoptosis was not considered so far. Even decreased apoptosis has been reported in thymocytes and erythrocytes upon IK1 inhibition [22], [23]. The particular new information of this manuscript is that the potassium channel inhibitor TRAM-34 not only decreases melanoma cell proliferation, but also efficiently enhances TRAIL-induced apoptosis via the mitochondrial pathway and is able to overcome TRAIL resistance of melanoma cells.

## Materials and Methods

### Cell Culture

Human melanoma cell lines enclosed TRAIL-sensitive (A-375, Mel-HO, SK-Mel-13, SK-Mel-28) and resistant cells (Mel-2a and MeWo) [7]. Subclones with induced TRAIL resistance (SK-Mel-13-TS, Mel-HO-TS, A-375-TS) derived from selection with 100 ng/ml TRAIL [8]. A-375 subclones stably transfected with a pIRES-Bcl-2 plasmid (A375-Bcl-2) or pIRES (A375-Mock) had been described previously [24]. Parental HCT-116 colon carcinoma cells were from ATCC (Maryland, MD, USA), and embryonic kidney cells (HEK-293) from DSMZ (Braunschweig, Germany). The HCT-116 Bax knockout, Bak knockdown and Bax/Bak double knockdown cells were kindly provided by B. Vogelstein (John Hopkins Cancer Center, Baltimore, MD, USA) [25]. Above cells were cultivated in DMEM (4.5 g/l glucose; Gibco, Invitrogen, Karlsruhe, Germany) with 10% FCS and antibiotics; HEK-293 furthermore received 1 mM pyruvate. SW480 colon carcinoma and HeLa cervix carcinoma cells (ATCC) were cultured in RPMI 1640 medium with L-glutamine (Biochrom, Berlin, Germany). Culture conditions were 37°C, 5% CO_2_. TRAIL-selected cells were continuously kept with 5 ng/ml TRAIL until 24 h before treatment. Cells were plated in 6-, 24- or 96-well plates with 2×10^5^, 5×10^4^ and 5×10^3^ cells, respectively, and treatment was started after 24 h.

For induction of apoptosis, the following agents were used: TRAIL (Alexis, Gruenberg, Germany, ALX-201-073-C020, 20 ng/ml); the selective IK1 inhibitor TRAM-34 (20–80 µM, kindly provided by H. Wulff, University of California, Davis, CA, USA) [20]; the IK1 inhibitor charybdotoxin (Sigma-Aldrich, Taufkirchen, Germany, 100–200 nM). For inhibition of apoptosis, the following agents were used: selective DR4/DR5 antagonistic antibodies (Alexis, ALX-804-297, ALX-804-298, 10 µg/ml; preincubation for 1 h); the pancaspase inhibitor Q-VD-OPh (MP Biomedicals, Ohio, USA; 10 µM, preincubation for 1 h).

### Plasmid and siRNA Transfection

Transient cell transfection was performed in 6-well plates at 24 h after seeding (70% confluence); TRAM/TRAIL treatment followed after another 24 h. An amount of 20 pmol siRNA and 4 µl TurboFect (Fermentas, St. Leon-Rot, Germany) per well were used. The siRNAs for SMAC (sc-36505), Bid (sc-29800) and the scrambled control (sc-37007) derived from Santa Cruz Biotechnology Inc. (Santa Cruz, CA, USA). For transient overexpression, cells were transfected for 4 h at 37°C with 2.5 µg/ml plasmid DNA and 0.1% DMRIE-C (Invitogen, Germany) in Opti-MEM medium (Life Technologies, Carlsbad, CA, USA) [26]. A pCMV-Sport6 plasmid with the full length sequence of human XIAP was used (IRATp970H0655D; Source BioScience imaGenes, Berlin, Germany). For IK1, a pcDNA plasmid with human IK1 [27] was kindly supplied by A. Schwab (University of Münster, Germany). The plasmid pcDNA3.1 (Invitogen, Germany) served as mock control.

### Electrophysiological Recordings

Potassium currents were recorded at 25°C in a whole-cell patch-clamp configuration [28], using an EPC10 amplifier and appropriate software (HEKA Elektronik, Germany). Series resistance errors were compensated in the range of 70–90%. Standard bath solution [mM]: 5 KCl, 155 NaCl, 2 CaCl_2_, 10 HEPES, pH 7.4. Internal pipette solution [in mM]: 130 KCl, 2 MgCl_2_, 10 EGTA, 10 HEPES, 9.3 CaCl_2_, pH 7.4. Under these conditions, intracellular Ca^2+^ was at 1 µM. Currents were induced by 500 ms voltage ramps, ranging from -100 to +50 mV. TRAM-34 (0.2 µM in standard bath solution) was directly applied in the cell vicinity. In time kinetics, mean currents were determined between –40 and –25 mV.

### Cell Proliferation, Apoptosis and Cytotoxicity

For proliferation analysis, cells were incubated for 4 h at 37°C with 10% of WST-1 reagent (Roche diagnostics, Mannheim, Germany), followed by determination of OD_450_ in an ELISA reader. Non-treated controls were defined as 100%. For continuously monitoring cell growth, the xCELLigence system (Roche diagnostics; Penzberg, Germany) was applied.

For quantification of apoptosis, cell cycle analyses were performed [29]. Cells harvested by trypsinisation were stained for 1 h with propidium iodide (Sigma-Aldrich, 200 mg/ml), and sub-G1 fractions were quantified by flow cytometry in a FACS Calibur (BD Bioscience, Bedford, USA). Cytotoxicity was determined in parallel by measuring LDH activity in culture fluids applying a cytotoxicity detection assay (Roche Diagnostics). Mitochondrial membrane potential was measured with the fluorescent dye TMRM^+^ (Tetramethylrhodamine methyl ester perchlorate; Sigma-Aldrich; 1 mM). Cells harvested by trypsinisation were stained for 15 min at 37°C and were analyzed by flow cytometry.

### Synergism Studies

For proving synergistic effects, melanoma cells were seeded in 24-well plates. Treatment started after 24 h with constant concentrations ratios of TRAM-34 (20–80 µM) and TRAIL (10–40 ng/ml). After 24 h of treatment, apoptosis was assessed by PI staining, and monotreatments were compared to combinations. Synergistic effects were proven by dose effect analyses resulting in normalized isobologram images, as commonly described [30]. Combination effects were evaluated by the combination index (CI), calculated according to the following equation: CI = D_1_/Dx_1_+D_2_/Dx_2_. Here, Dx_1_ and Dx_2_ represent concentrations of TRAIL and TRAM-34 needed to produce a certain apoptosis effect when given alone, whereas D_1_ and D_2_ are the concentrations needed for the apoptosis effect in the combination. Synergistic effects are characterized by CI values of <1, whereas a CI of 1 indicates additive effects [30].

### Expression Analyses

For mRNA analysis, RNA was extracted with a NucleoSpin RNA kit (Macherey-Nagel, Düren, Germany), and first strand cDNA was produced with a kit of Fermentas (St. Leon-Roth, Germany). Real-time PCR with 35 cycles (10 s at 95°C, 30 s at 60°C) in SYBR Green PCR master mix (Qiagen, Hilden, Germany) was performed in a Masterplex thermocycler (Eppendorf, Wesseling-Berzdorf, Germany). Primers for IK1 and β-actin were from Qiagen (QuantiTect QT00003780 and QT00095431). Relative IK1 expression was quantified according to the ΔCT method using β-actin for normalization [31].

For analysis of receptor surface expression, cells harvested with EDTA were stained with monoclonal antibodies against TRAIL receptors (DR4, ALX-804-297; DR5, ALX-804-298, Alexis; DcR1, sc-65308; DcR2, sc-65311, Santa Cruz; 1∶100). Mouse IgG1 (Ancell, # 278-010) served as control. After incubation with a secondary antibody (Alexis, ALX 211-201, 1∶100, 30 min), surface expression was quantified by flow cytometry. SW480 and HeLa cells served as positive controls for decoy receptors.

For Western blotting, total protein extracts were obtained by cell lysis in 150 mM NaCl, 1 mM EDTA, 2 mM PMSF, 1 mM leupeptin, 1 mM pepstatin, 0.5% SDS, 0.5% NP-40 and 10 mM Tris-HCl (pH 7.5). Cytosolic and mitochondrial cell fractions were obtained by a kit of PromoKine (Heidelberg Germany). Western blotting on nitrocellulose membranes was described previously [26].

Primary antibodies from Cell Signaling: cleaved caspase-3 (rabbit, 1∶5000), caspase-3 (rabbit, 1∶1000), caspase-8 (mouse, 1∶1000), caspase-9 (rabbit, 1∶1000), XIAP (rabbit, 1∶1000), PARP (rabbit, 1∶1000). Primary antibodies from Santa Cruz: Mcl-1 (mouse, 1∶200), Bcl-2 (mouse; 1∶500; rabbit; 1∶200), Bax (rabbit, 1∶200), Bak (rabbit, 1∶500), Bid (rabbit, 1∶1000), cIAP2 (rabbit, 1∶1000), survivin (mouse, 1∶500), AIF (goat, 1∶1000), SMAC (mouse, 1∶1000), IK1 (H-120, rabbit, 1∶1000), GAPDH (mouse, 1∶1000). Further antibodies: PUMA (Epitomics, rabbit, 1∶1000), Noxa (Pro Sci, rabbit, 1∶500), DR4 (Abcam, rabbit, 1∶500), DR5 (Abcam, rabbit, 1∶500), cytochrome c (BD Biosciences, mouse, 1∶1000), VDAC (Calbiochem; mouse; 1∶5000), prohibitin (Thermo Scientific, mouse, 1∶1000). Secondary antibodies from Dako (Hamburg, Germany; 1∶5000): peroxidase-labeled goat anti-rabbit, goat anti-mouse, rabbit anti-goat antibodies.

### Statistical Analyses

Assays consisted of triplicates, and at least two independent experiments were performed. Mean values and SDs were calculated by enclosing all individual values of the independent experiments (at least 6 values). Statistical significance was proven by Student’s t-test (normal distribution) or Wilcoxon test (non-normal distribution of data), and p-values of <0.05 were considered statistically significant.

## Results

### Antiproliferative Effects of TRAM-34

**Figure 1:**
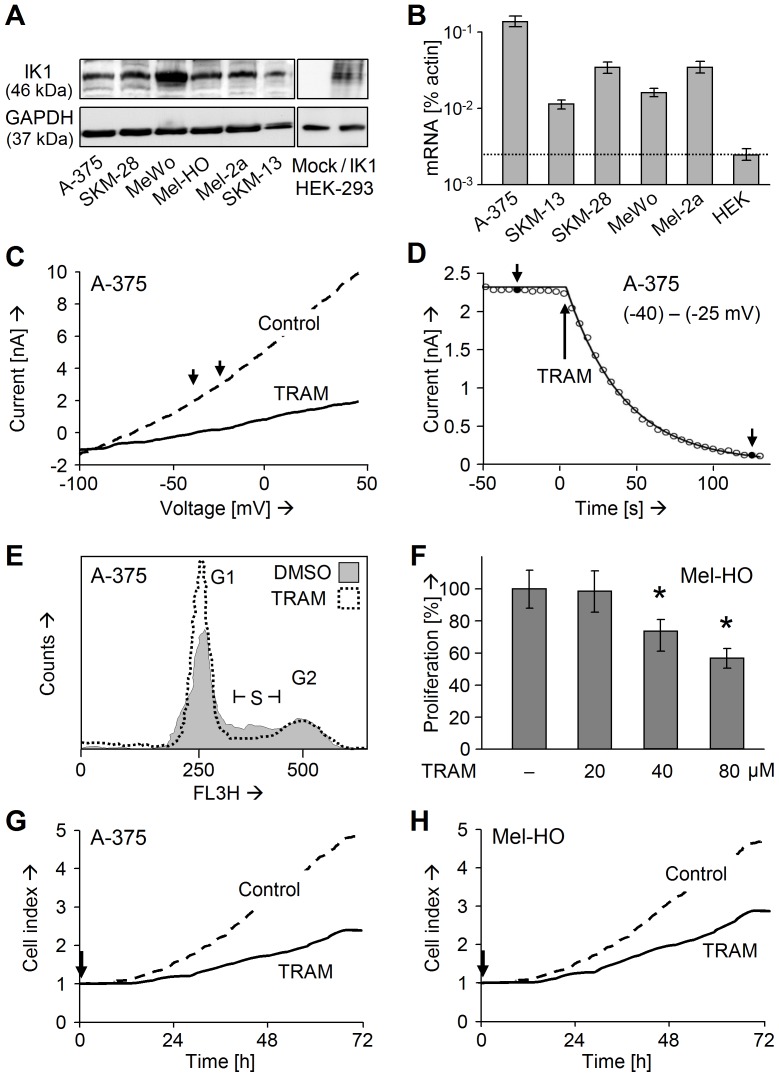
Antiproliferative effects by IK1 inhibition. (A) Protein expression of IK1 is shown in six melanoma cell lines. Negative control: mock-transfected HEK-293; positive control: IK1-transfected HEK-293 cells. Equal protein loading (30 µg per lane) is proven by GAPDH. (B) Expression of IK1 mRNA in melanoma cell lines was determined by real-time PCR. The threshold of negative HEK-293 is indicated. Values were normalized to β-actin. (A, B) Each two independent experiments revealed comparable results. (C) Voltage-dependent potassium currents were recorded in TRAM-34-treated A-375, as compared to non-treated controls. The voltage range, used for subsequent time course, is indicated. (D) Time course of potassium currents in A-375 after addition of TRAM-34 (t = 0) is shown. Short arrowheads indicate the time interval for determination of the current/voltage dependency. (E) Cell cycle analysis of TRAM-34-treated A-375 as compared to DMSO-treated controls. (F) Decreased proliferation in response to increasing concentrations of TRAM-34 is shown for Mel-HO, as determined by WST-1 assay. Means and SDs are shown of three independent experiments, each one consisting of triplicates. Statistical significance is indicated (*; p<0.005), when comparing TRAM-34-treated cells with DMSO-treated controls. (G, H) Real-time growth curves of TRAM-34-treated A-375 and Mel-HO are compared to DMSO-treated controls. Cell indices were normalized at t = 0.

Significant expression of the potassium channel IK1 (46 kDa) was observed in a panel of six melanoma cell lines by Western blotting. HEK-293 cells were negative, and mock-transfected cultures were used as negative controls. The specificity of the IK1 antibody was controlled by transient transfection with an IK1 plasmid, which restored expression of the 46/47 kDa protein band in HEK-293 (Fig. 1A). Also expression of IK1 mRNA was proven in melanoma cell lines by real-time PCR, whereas HEK-293 remained negative (Fig. 1B).

For demonstrating functional IK1 in melanoma cells, linear potassium currents were recorded in response to a voltage ramp protocol in A-375 under conditions of 1 µM intracellular Ca^2+^. Application of TRAM-34 reduced the current from 10 nA to 2 nA, suggesting IK1 as the dominant potassium channel in A-375 (Fig. 1C). Monitoring the time course of potassium currents at voltage conditions between −40 mV and −25 mV revealed a rapid and immediate decrease upon TRAM-34 treatment (Fig. 1D).

Antiproliferative effects of TRAM-34 on melanoma cells were determined by cell cycle analysis. Treatment with 40 µM TRAM-34 for 24 h resulted in a partial G1 arrest and diminished S-phase (Fig. 1E). Decreased dose-dependent cell proliferation was seen for Mel-HO and A-375, as determined by WST-1 assay (Fig. 1F; data not shown). Real-time cell analysis revealed continuous antiproliferative effects by TRAM-34, which started immediately after addition and resulted in a decrease of 2-3-fold at 72 h (Fig. 1G, 1H).

### Sensitization of Melanoma Cells for TRAIL-induced Apoptosis

**Figure 2:**
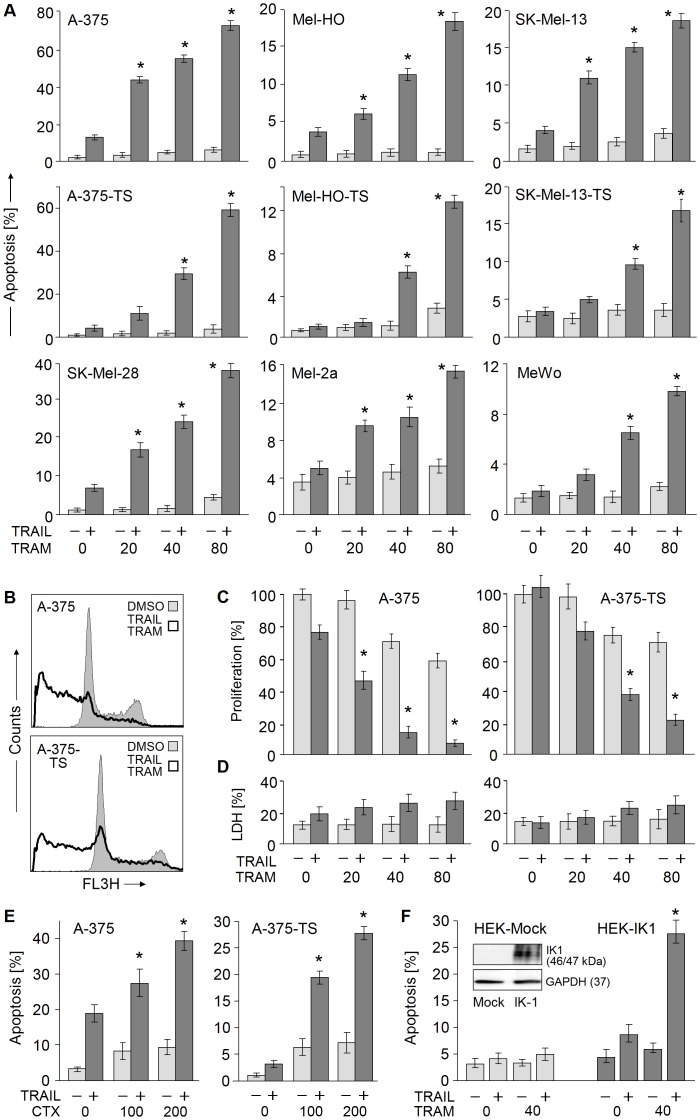
Sensitization of melanoma cells for TRAIL-induced apoptosis. **(**A) Apoptosis (percentage of sub-G1 cells) was determined by cell cycle analyses in nine melanoma cell lines treated with increasing concentrations of TRAM-34+/− TRAIL. (B) Examples are shown of A-375 and A-375-TS treated with the combination of 40 µM TRAM-34+ TRAIL (open graph) as compared to DMSO-treated controls (filled graph). (C, D) For A-375 and A-375-TS, cell proliferation was determined by WST-1 (C) and cytotoxicity by LDH-release (D). (E) Apoptosis was monitored after treatment with two concentrations of charybdotoxin (CTX) +/− TRAIL. (F) Induction of apoptosis by TRAM-34+/− TRAIL was determined in mock- and IK1-transfected HEK-293 cells. Protein expression of IK1 at 24 h after transfection is shown in the inset, as determined by Western blotting. (A, C, D, E, F) Means and SDs are shown of three independent experiments, each one consisting of triplicates. Statistical significance (*; p<0.005) is indicated, when comparing TRAM/TRAIL-treated cells with TRAIL-treated cells.

For investigating strategies to overcome TRAIL resistance in melanoma, a panel of TRAIL-sensitive and resistant melanoma cell lines was studied. In addition, cell populations of A-375, SK-Mel-13 and Mel-HO were used, which had been selected for TRAIL resistance. Despite its antiproliferative activity (Fig. 1G), TRAM-34 alone (20–80 µM) remained largely without effect on apoptosis at 24, 48 and 72 h (Fig. 2A; data not shown). In combination with TRAIL, however, TRAM-34 resulted in strong enhancement of apoptosis in all sensitive and resistant cell populations. Thus in TRAIL-sensitive A-375, Mel-HO, SK-Mel-28 and SK-Mel-28 cells, apoptosis was induced by TRAM/TRAIL in 18%–70% of cells corresponding to a factor of >4-fold, as compared to TRAIL alone (Fig. 2A, 2B).

Also selected TRAIL-resistant cells (A-375-TS, Mel-HO-TS, SK-Mel-13-TS) were highly responsive, resulting in apoptotic rates between 13% and 60%, and even cell lines with pre-existing TRAIL resistance (MeWo, Mel-2a) responded with 10% and 15% apoptosis, respectively (Fig. 2A). Apoptosis appeared as the dominant effect, as cytotoxicity remained rather unaffected at 24 h and at later times, as determined by LDH release (Fig. 2D; data not shown). The proapoptotic effects of TRAM/TRAIL resulted in strongly reduced cell proliferation, as determined by WST-1 assay in A-375 and A-375-TS. Whereas TRAIL alone decreased cell proliferation by 25% in A-375 and remained without effect on A-375-TS, TRAM/TRAIL resulted in a reduction of 95% and 75%, respectively (Fig. 2C).

For proving that enhanced apoptosis was mediated via IK1, the alternative IK1 inhibitor charybdotoxin (CTX) was applied. Comparable to TRAM-34, CTX (100–200 nM) only weakly induced apoptosis in A-375 and A-375-TS but strongly enhanced TRAIL sensitivity (Fig. 2E). As a final proof for the critical role of IK1 in this setting, mock-transfected, IK1-negative HEK-293 cells did not respond to TRAM/TRAIL, whereas strongly enhanced apoptosis (27%) was obtained after transient transfection and overexpression of IK1 (Fig. 2F).

**Figure 3:**
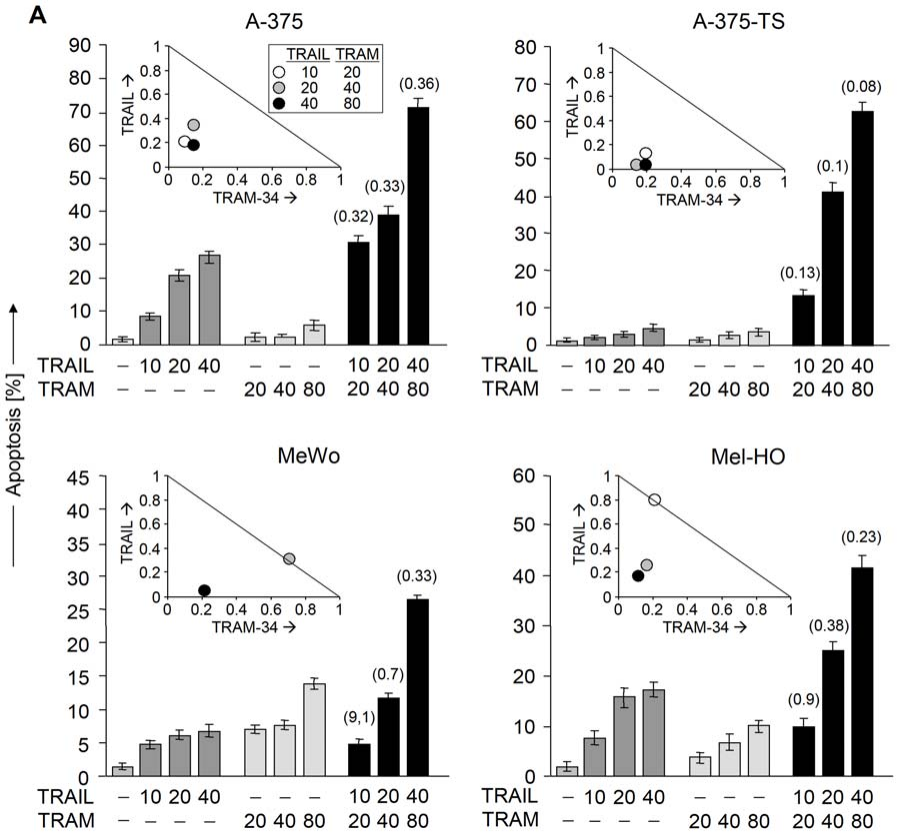
Synergism studies for TRAM-34 and TRAIL. (A) Induction of apoptosis (sub-G1 cells) is shown in four melanoma cell lines in response to TRAM-34 (20–80 µM) and TRAIL (10–40 ng/ml), given alone or in combination. Calculated CI values are given in parenthesis above the bars of the combinations. For dose effect analyses, relative concentrations of TRAM-34 were plotted against relative concentrations of TRAIL shown in normalized isobolograms (insets). The hypotenuse indicates the line of additive effects, whereas values below are synergistic. Three independent experiments revealed largely similar results.

Enhancement of TRAIL-induced apoptosis by TRAM-34 appeared as clearly synergistic, as proven by dose effect analyses in A-375, A-375-TS, MeWo and Mel-HO. Thus keeping constant concentration ratios, combinations of TRAIL (10–40 ng/ml) and TRAM-34 (20–80 µM) were applied, and the effects of the combination and single treatments were compared. In this way, synergistic effects were proven for all three combinations in A-375 and A-375-TS (CI values <0.4), for the two higher concentrations in Mel-HO and for the highest concentration of 40 ng/ml TRAIL/80 µM TRAM-34 in MeWo (CI = 0.33; Fig. 3).

### Upregulation of TRAIL Receptors

**Figure 4:**
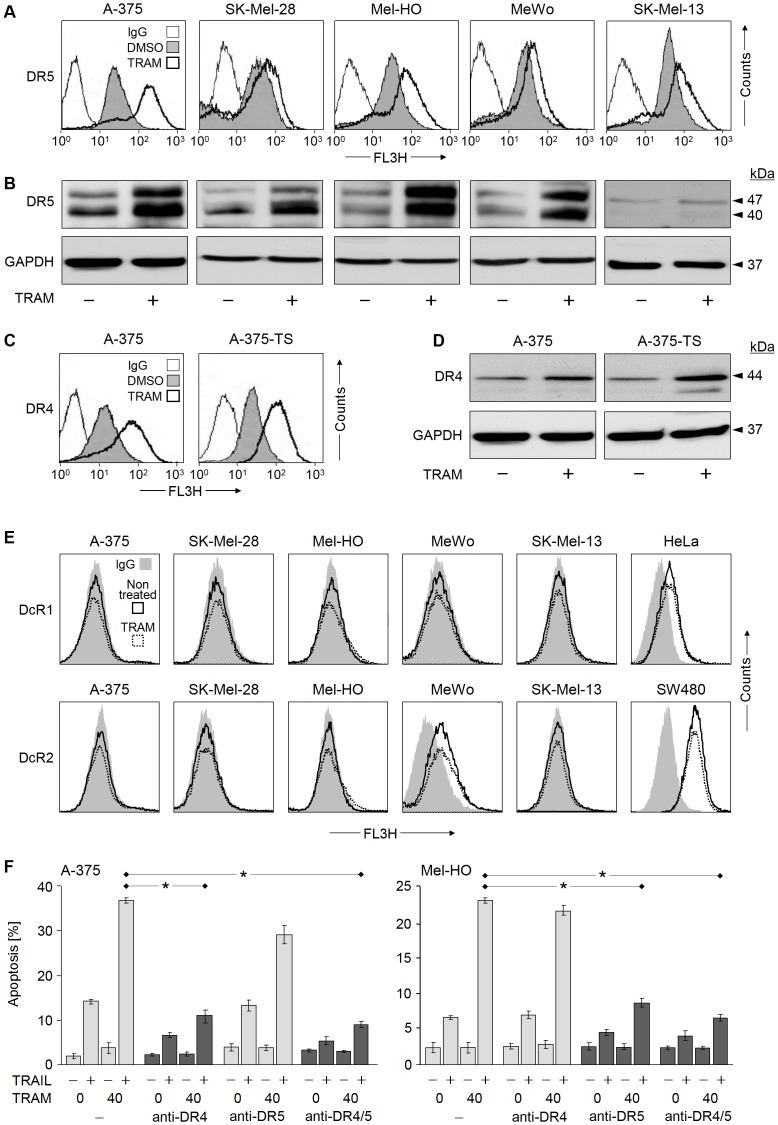
Death receptor expression in response to TRAM-34. (A, B) Expression of DR5 is shown in five melanoma cell lines in response to TRAM-34 (40 µM, +), and (C, D) expression of DR4 is shown in A-375 and A-375-TS, as compared to DMSO-treated controls (−). Total protein expression was determined by Western blotting, and surface expression was determined by flow cytometry. (E) Expression of DcR1 and DcR2 is shown by flow cytometry in five melanoma cell lines in response to TRAM-34. HeLa and SW480 cells served as positive controls for DcR1 and DcR2, respectively. IgG1-stained cells served as negative controls. Three (A, C) or two (E) independent experiments, with each time triplicates revealed highly similar results. (F) Apoptosis in response to TRAM/TRAIL was monitored in A-375 and Mel-HO after blocking DR4 and/or DR5 by selective antagonistic antibodies (mean values and SDs of two independent experiments, each one with triplicates). Statistical significance is indicated (*; p<0.005), when comparing combined treatment with TRAIL alone.

In agreement with the critical importance of TRAIL receptor expression levels for melanoma cell sensitivity to TRAIL [7], significant upregulation of DR4 and DR5 was seen in response to TRAM-34. Thus DR5, which is expressed in all melanoma cell lines, was generally upregulated at the levels of total cellular protein and surface expression (Fig. 4A, 4B). Similarly, DR4 which is expressed by A-375 and A-375-TS was upregulated in these cells (Fig. 4C, Fig. 4D). No basal surface expression of TRAIL decoy receptors DcR1 and DcR2 was found in A-375, SK-Mel-28, Mel-HO and SK-Mel-13, as compared to the controls HeLa and SW480. Only in MeWo, some expression of DcR2 was seen. The treatment with TRAM-34, however, remained without effect on decoy receptor expression in melanoma cells (Fig. 4E).

For investigating the specific contribution of these receptors to TRAM/TRAIL-induced apoptosis, selective antagonistic antibodies were applied. Clearly, apoptosis in DR4(+) A-375 was preferentially blocked by the DR4 antagonist (70%), whereas apoptosis in DR4(−) Mel-HO was largely blocked by the DR5 antagonist (70%) (Fig. 4F). These experiments proved that TRAM-34 specifically enhanced TRAIL-induced apoptosis pathways via the TRAIL receptors.

### Involvement of Caspases

**Figure 5:**
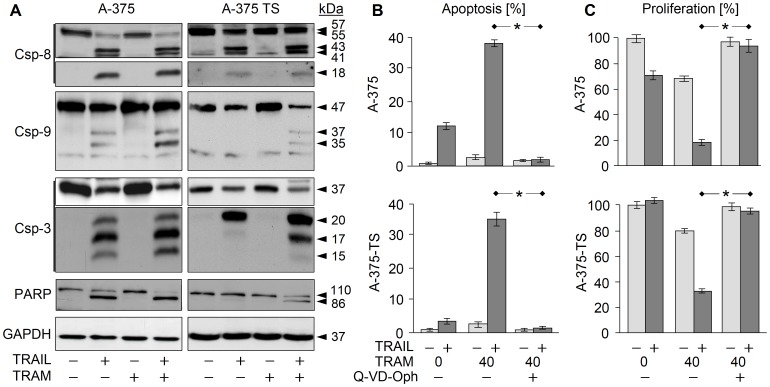
Caspase activation in response to TRAM and TRAIL. (A) Processing of caspase-8, -9 and -3 in A-375 and A-375-TS in response to TRAM-34 (40 µM) and TRAIL is shown by Western blotting. Two independent experiments revealed comparable results. (B) Apoptosis (% of sub-G1 cells) in A-375 and A-375-TS is shown in response to TRAIL, TRAM-34 and the pancaspase inhibitor Q-VD-OPh (10 µM, 1 h pretreatment). (C) Cell proliferation was determined by WST-1. (B, C) Mean values and SDs are shown of two independent experiments, each one with triplicates. Statistical significance of abrogated apoptosis and recovered cell proliferation by Q-VD-OPh is indicated (*; p<0.005).

In sensitive A-375, TRAIL alone already resulted in significant activation of the caspase cascade, seen by cleavage products of caspase-8 (18, 41, 43 kDa), caspase-9 (35, 37 kDa) and caspase-3 (15, 17, 20 kDa). Paradoxically, processing of caspase-8 and caspase-3 was not further enhanced by the combination, whereas some enhancement was seen for caspase-9 (Fig. 5A). Also in A-375-TS, TRAIL alone resulted in processing of caspase-8 and of caspase-3 to a 20 kDa premature product. Again, processing of caspase-8 was not enhanced by the combination. Upon combined treatment, however, caspase-9 processing and full processing of caspase-3 to its mature cleavage products (15, 17 kDa) was seen. Further proving enhanced caspase-3 activity, stronger cleavage of the death substrate PARP (86 kDa) appeared in response to combination treatment (Fig. 5A). Thus, mechanisms were suggestive that enhanced activation of caspase-9 in both cell lines and enabled full processing of caspase-3 in A-375-TS. The role of caspases was also proven by the pancaspase inhibitor Q-VD-OPh, which completely prevented TRAM/TRAIL-induced apoptosis and restored cell viability (Fig. 5B; 5C).

### Dependency on Mitochondria, Bax and Bcl-2

**Figure 6:**
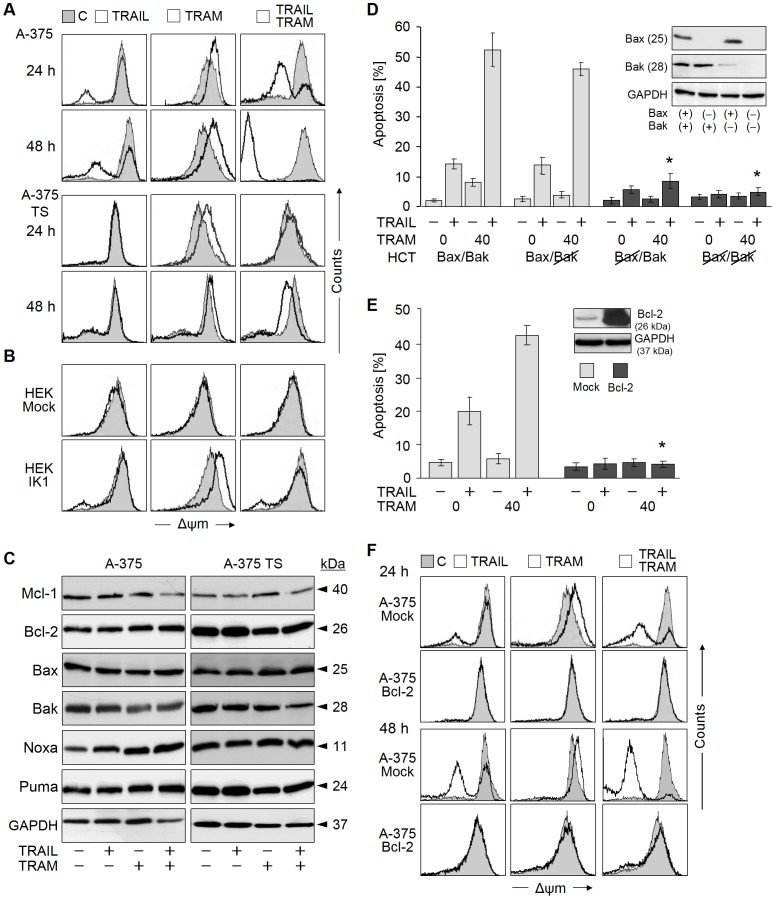
Dependency on Bax and Bcl-2. (A, B, F) Mitochondrial membrane potential (Δψm) was measured in A-375, A-375-TS, mock and IK1-transfected HEK-293, A375-Mock and A375-Bcl-2 at 24 h and 48 h of treatment with TRAM-34 (40 µM) +/− TRAIL. Treated cells (open graphs) were compared with DMSO-treated controls (grey). Each three independent experiments showed comparable results. (C) Expression of Bcl-2 proteins was determined by Western blotting in A-375 and A-375-TS in response to TRAM-34 (40 µM) +/− TRAIL. Two independent experiments showed comparable results. (D, E) Apoptosis induced by TRAM/TRAIL was investigated in (D) HCT-116 parental cells (Bax^+^, Bak^+^) and in subclones with knockdown for Bax and/or Bak, as well as in (E) A-375 cells stably transfected with Bcl-2 (A375-Bcl-2) and mock transfected controls. Expression of Bcl-2 proteins was controlled by Western blotting (insets). Statistical significance is indicated (*; p<0.005) for comparison of parental cells and mock controls.

An involvement of mitochondrial apoptosis pathways was evident in A-375 due to an enhanced decrease of the mitochondrial membrane potential Δψm, as compared to TRAIL alone (24 h: 22% → 56%; 48 h: 40% → 90%). TRAM-34 however did not decrease Δψm by itself, rather it resulted in a hyperpolarization shown for A-375 and A-375-TS at 4, 24 and 48 h (Fig. 6A; data not shown). This suggested a direct effect of TRAM-34 on mitochondria. In A-375-TS, there was no decrease of Δψm by TRAIL indicating a block at the mitochondrial level. Under combination treatment, however, the hyperpolarization of TRAM-34 was shifted back at 24 h and even below the control peak at 48 h (Fig. 6A).

The TRAM-34-mediated increase of Δψm was clearly dependent on IK1, as seen in mock-transfected and IK1-transfected HEK-293. Whereas there was no increase of Δψm in mock-transfected cells in response to TRAM-34, the effect was restored by IK1 transfection. Again, the hyperpolarization was shifted back by the combination with TRAIL (Fig. 6B).

The effects on mitochondria were not related to any changes at the expression level of Bcl-2 proteins, as proven for a panel of pro- and anti-apoptotic factors (Fig. 6C). TRAM/TRAIL-mediated apoptosis was however clearly dependent on Bax, and it was abrogated by Bcl-2 overexpression. Thus, in Bax^+^/Bak^+^ HCT-116 colon carcinoma cells, TRAIL-induced apoptosis was similarly enhanced by TRAM-34 (14% → 50%). Apoptosis was almost completely abrogated in Bax knockout and in Bax/Bak double knockdown cells, whereas Bak knockdown had only little effect (Fig. 6D). Similarly, stable overexpression of Bcl-2 (A375-Bcl-2) completely protected against the proapoptotic effects of TRAM/TRAIL (Fig. 6E). Both, loss of Δψm by combination treatment and membrane hyperpolarization by TRAM-34, were completely blocked by Bcl-2 overexpression (Fig. 6F). The experiments proved the critical role of mitochondria for TRAM-34 proapoptotic effects.

### Release of Mitochondrial Factors

**Figure 7:**
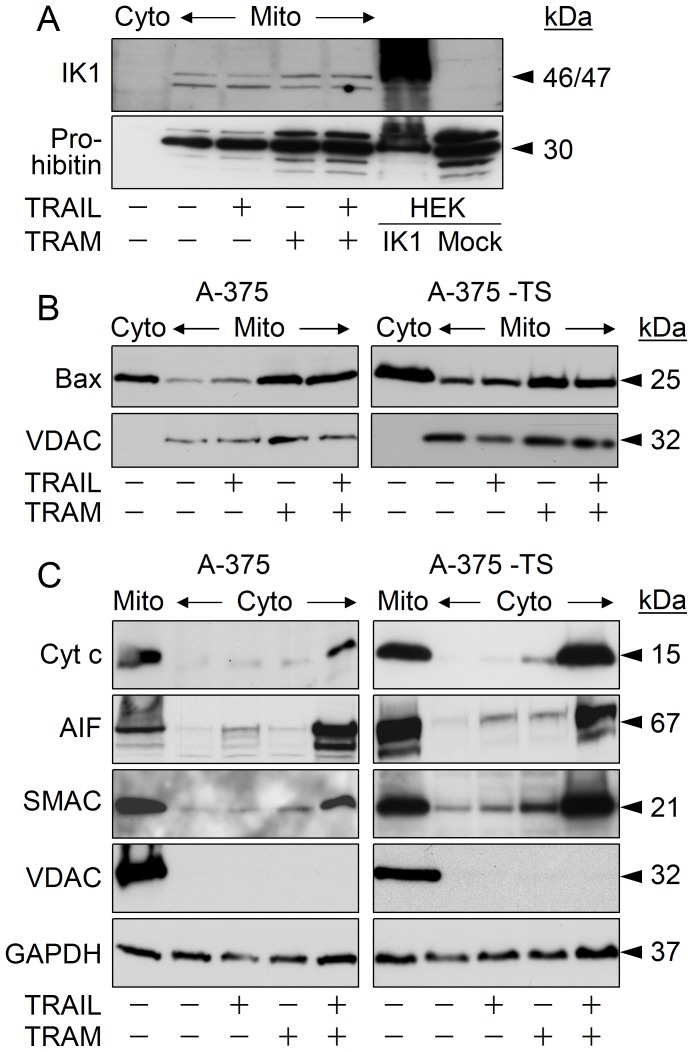
Mitochondrial release of proapoptotic factors. (A) Mitochondrial extracts of A-375, treated as indicated with TRAM-34 and TRAIL for 24 h, were analyzed for IK1 by Western blotting. Comparable amounts of mitochondrial extracts were loaded as proven by incubation with prohibitin antibody. A cytosolic extract (Cyto) as well as total protein extracts of mock or IK1-transfected HEK-293 served as controls. (B, C) Mitochondrial (Mito) and cytosolic extracts (Cyto) of A-375 and A-375-TS treated for 4 h with TRAM-34 (40 µM) +/−TRAIL were investigated by Western blotting. Equal loading was proven by the mitochondrial protein VDAC and cytosolic protein GAPDH, respectively. VDAC applied to cytosolic extracts ruled out contaminations with mitochondria.

Direct effects of TRAM-34 on mitochondria could be explained by the presence of IK1 in mitochondrial membranes. Indeed, significant IK1 expression was seen in mitochondrial extracts, which remained unchanged upon treatment (Fig. 7A). In agreement with its Bax dependency (Fig. 6D), mitochondrial translocation of Bax was seen in A-375 and A-375-TS in response to TRAM-34 alone or combination treatment. This happened at 4 h, thus before any apoptosis was induced, indicative for an initial step (Fig. 7B).

Of particular note was the strong release of mitochondrial factors as cytochrome c, AIF and SMAC already at 4 h of combination treatment (Fig. 7C). The same response was seen in A-375, in A-375-TS and A-375-Mock at 24 h of treatment, whereas the release of mitochondrial factors was prevented by Bcl-2 overexpression in A375-Bcl-2 (data not shown).

**Figure 8:**
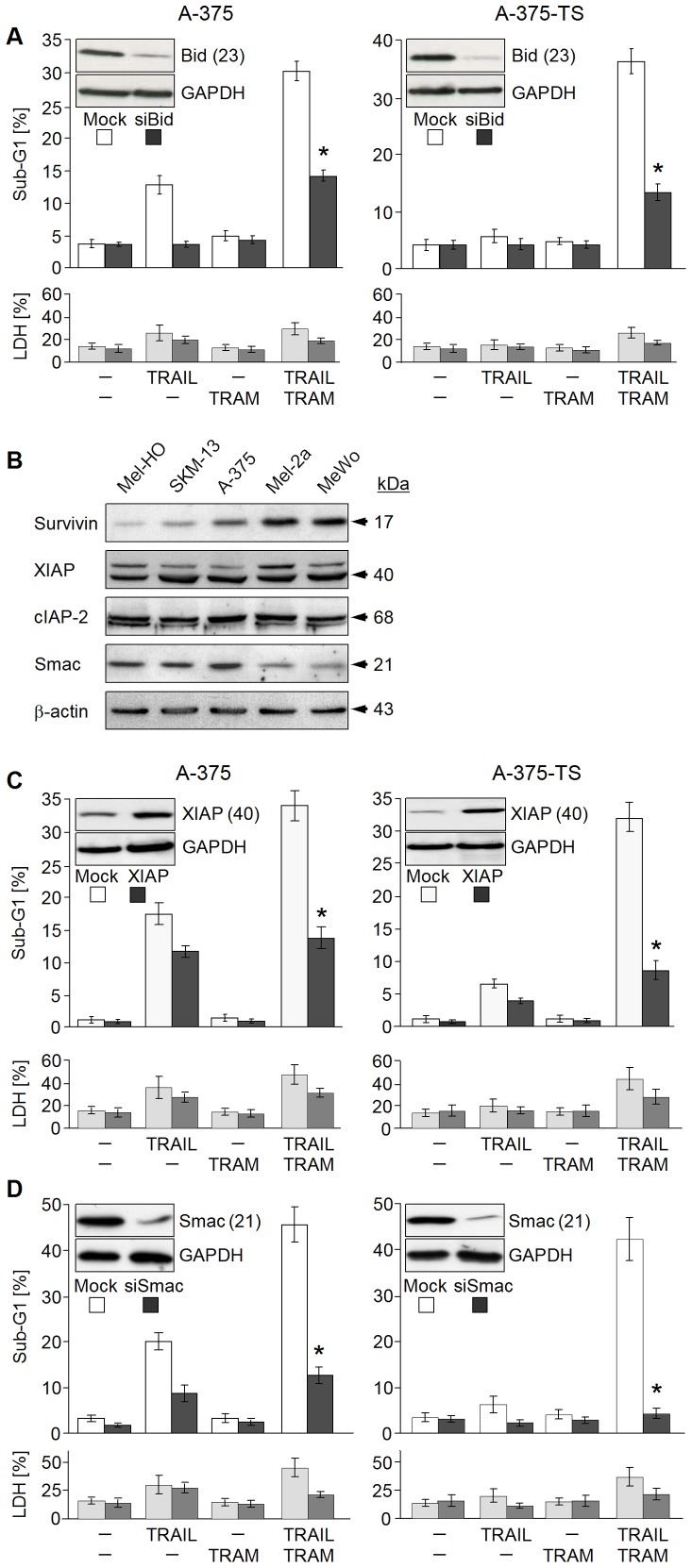
Dependency on XIAP, Bid and SMAC. (A, C, D) The sensitivity of A-375 and A-375-TS cells for TRAM/TRAIL-induced apoptosis is shown (A) after siRNA-mediated Bid knockdown, (C) plasmid-mediated XIAP overexpression and (D) siRNA-mediated SMAC knockdown. The respective mock controls are shown for comparison. Apoptosis (% of sub-G1 cells) and cytotoxicity (LDH release) is shown at 24 h of treatment. Mean values and SDs of three (XIAP, SMAC) or two independent experiments (Bid) are shown; each experiment consisted of triplicates. Statistical significance is indicated (*; p<0.005), when comparing to the respective mock controls. Overexpression or downregulation is shown in insets. (B) Expression of cIAPs (survivin, XIAP and cIAP-2) as well as of SMAC was analyzed by Western blotting in total protein extracts of melanoma cell lines.

Death receptor signalling and mitochondrial pathways are interconnected through the proapoptotic Bcl-2 protein Bid, which is activated by caspase-8. We investigated its role in TRAM/TRAIL-induced apoptosis, by applying anti-Bid siRNA in A-375 and A-375-TS. Bid knockdown strongly diminished apoptosis by two to threefold (Fig. 8A), thus proving the dependency on the mitochondrial amplification loop and again underlining, that specific TRAIL pathways were enhanced (Fig. 8A).

### The Decisive Role of the SMAC-cIAP Rheostat

According to caspase analysis (Fig. 5A), inhibited caspase-3 appeared as limiting TRAIL-induced apoptosis. This was an indication for the antiapoptotic function of cIAPs. Melanoma cell lines revealed significant expression of cIAPs as survivin, cIAP-2 and XIAP as well as of the cIAP antagonist SMAC (Fig. 8B). For investigating the role of XIAP, it was overexpressed by plasmid transfection in A-375 and in A-375-TS. Proving the potency of XIAP as a major inhibitor of TRAIL-induced apoptosis in melanoma cells, its overexpression strongly diminished TRAM/TRAIL-induced apoptosis by 60–70%, as compared to mock-transfected cells (Fig. 8D).

In light of the strong release of mitochondrial AIF, cytochrome c and SMAC (Fig. 7C), the question was which of these factors could provide enhanced TRAM/TRAIL-induced apoptosis. Clearly supporting the decisive role of SMAC for overcoming inducible TRAIL resistance, its siRNA-mediated downregulation completely protected A-375-TS against the proapoptotic effects of TRAM/TRAIL (Fig. 8D). Also in parental A-375, SMAC knockdown strongly limited apoptosis to the level induced by TRAIL itself (Fig. 8C, 8D). Cytotoxicity was largely unaffected by these treatments at 24 h (Fig. 8A, 8C, 8D). Thus, the enhancement of TRAIL-induced apoptosis by TRAM-34 was essentially based on the release of SMAC. The rheostat of released SMAC and antiapoptotic cIAPs appeared as a critical level regulating TRAIL sensitivity in melanoma cells.

## Discussion

The death ligand TRAIL appears as a promising antitumor strategy, due to apoptosis induction in cancer cells, while normal cells were largely spared [32], [33]. However, clinical trials with TRAIL or TRAIL receptor agonists have shown only limited response so far in solid tumors [34], [35]. Insufficient efficacy may be attributed to inducible resistance, as has been seen in different tumor models, also in melanoma cells [8], [36]. Sensitization thus appears as an important step for establishing TRAIL-based therapies, and multiple strategies have been applied to enhance melanoma cell sensitivity to TRAIL [37]–[40].

A completely new strategy is presented here that is based on selective inhibition of the calcium-dependent potassium channel IK1, which has been reported as upregulated in several types of human cancers [21], [41], [42]. Also in melanoma cells, we have previously demonstrated IK1-dependent transmembrane potassium currents [28]. Here we prove IK1 mRNA and protein expression in a series of melanoma cell lines, thus suggesting IK1 expression as a characteristic feature of melanoma.

Inhibitors of IK1 are represented by clotrimazole and the scorpion venom charybdotoxin. The clotrimazole analogue TRAM-34 lacks non-specific inhibition of cytochrome P450, which is a clotrimazole side effect and associated with liver toxicity [20]. The specificity of TRAM-34-mediated effects via IK1 was proven here in IK1-negative HEK-293 cells, which did not respond, whereas IK1-transfected cells recovered responsiveness. IK1 has been related to aberrant tumor cell proliferation and migration [19], [43], [44], and antiproliferative effects upon IK1 inhibition were reported in different tumor cells [41]. In agreement, we show significant growth inhibition by TRAM-34 in melanoma cells.

Induction of apoptosis has so far not been related to IK1 inhibition. Rather, calcimycin-induced apoptosis was suppressed in lymphocytes by charybdotoxin and clotrimazole [22], and TRAM-34 suppressed the proapoptotic effects of cisplatin in epidermoid cancer cells [42]. However, combinations with death ligands had not been considered so far. In agreement with previous reports, TRAM-34 itself did not trigger apoptosis in melanoma cells. However, the combination with TRAIL synergistically enhanced apoptosis, and both pre-existing and inducible TRAIL resistance were overcome. Of particular note, all of nine melanoma cell lines were responsive, indicating a general mechanism that was targeted by TRAM-34. Thus, the relation of potassium channels to apoptosis regulation appears in a new light.

As concerning the mechanisms how TRAM-34 may enhance TRAIL-induced apoptosis, an upregulation of both agonistic TRAIL receptors was seen. This appears of particular interest, as downregulation of TRAIL receptors played an important role in inducible TRAIL resistance of melanoma cells [8], which is thus crossed by TRAM-34. Upregulation of TRAIL receptors has also been seen with resveratrol and indirubin [37], [40]. In contrast, TRAIL decoy receptors DcR1 and DcR2 appeared of no role in TRAM-34/TRAIL-induced apoptosis.

Remarkably however, processing of initiator caspase-8 was not significantly enhanced by combined TRAM/TRAIL treatment as compared to TRAIL alone, indicating the critical role of downstream regulation steps. Indeed, significant activation of mitochondrial apoptosis pathways was seen, as decreased mitochondrial membrane potential and release of proapoptotic mitochondrial factors (cytochrome c, AIF and SMAC). This mitochondrial activation appeared as essential for TRAM/TRAIL-induced apoptosis. Firstly, it preceded apoptosis induction, and secondly, apoptosis was strongly dependent on Bcl-2 proteins. Thus Bax knockout or Bcl-2 overexpression or siRNA-mediated knockdown of Bid strongly diminished TRAM/TRAIL-induced apoptosis. The critical role of mitochondrial pathways for TRAIL sensitization of melanoma cells has also been seen for indirubin and UVB [39], [40], thus underlining their importance for apoptosis induction in melanoma.

The question was how TRAM-34 could activate mitochondrial pathways. Potassium channels have mainly been considered as located in the cytoplasma membrane and controlling the membrane potential [19]. However, potassium conductance has also been identified across the mitochondrial membrane, which was mediated in lymphocytes by the voltage-gated potassium channel Kv1.3 [45]. Similarly, IK1 activity has recently been identified by patch clamp analysis in mitochondria of colon carcinoma cells [46]. Clearly supporting these findings, we show here significant IK1 expression in mitochondrial fractions of melanoma cells.

The mitochondrial activity of IK1 was clearly visible by hyperpolarization of Δψm in response to TRAM-34. This effect was clearly proven as dependent on IK1, as IK1-negative cells did not respond, but hyperpolarization was re-established after IK1 transfection. A similar effect on Δψm hyperpolarization was observed upon Kv1.3 inhibition preceding apoptosis [47], suggesting this step as characteristic for apoptosis mediated via potassium channels. This hyperpolarization effect may result from the role of the mitochondrial potassium influx in antagonizing increased Δψm by proton export [48].

Strongly relating potassium channels with the regulation of apoptosis, an physical interaction between Bax and Kv1.3 was reported in lymphocytes, which appeared as necessary for Bax-induced apoptosis [47]. Supporting the critical role of Bax, we see here a clear dependency of TRAM/TRAIL-induced apoptosis on Bax as well as mitochondrial translocation of Bax in response to TRAM-34, as an early effect. Thus the repertoire of IK1 activities has to be extended by the induction of mitochondrial apoptosis pathways.

Coming back to the roles of mitochondrial factors released in course of TRAM/TRAIL-induced apoptosis, caspase-independent AIF [15] may play a less important role, as apoptosis was abrogated by caspase inhibitors. Cytochrome c, which activates caspase-9 [49], may contribute to the sensitization of A-375-TS and A-375. Most suitable for explaining enhanced apoptosis by TRAM-34 appeared a rheostat of antiapoptotic cIAPs and released SMAC.

Proteins of the IAP family are frequently overexpressed in cancer cells and have been implicated in tumor growth, pathogenesis and chemoresistance [50].

To prevent cell death, XIAP directly binds to processed caspase-9 and caspase-3 [51]. Also melanoma cells revealed significant expression of cIAPs, suggesting that this was the brake, which prevented full caspase-3 activation in response to TRAIL. The hypothesis was that SMAC release induced by TRAM/TRAIL resulted in XIAP neutralization and release of caspase-3 activity. The hypothesis was proven by SMAC knockdown, which completely abrogated TRAM/TRAIL-induced apoptosis in TRAIL-resistant A-375-TS. Some remaining apoptosis in parental A-375 may depend on a residual direct caspase cascade in these cells.

The important role of the SMAC/XIAP rheostat has also been addressed in other studies leading to the development of small molecule XIAP inhibitors to enhance TRAIL sensitivity [51], [52]. Its decisive role for TRAM/TRAIL-induced apoptosis in melanoma cells was further supported by XIAP overexpression, which diminished apoptosis by 50%. The particular function of TRAM-34 in this setting was the opening of the mitochondrial pathway through Bax activation. The finding that XIAP overexpression did not completely prevent TRAM/TRAIL-induced apoptosis furthermore indicated the presence of a master regulator, which was able to undermine XIAP activity.

In view of development of effective therapies for melanoma, TRAM-34 and TRAIL may be considered. Both TRAIL and clotrimazole analogues have been seen in clinical trials with only minor side effects reported [34], [53]. Due to the data in this manuscript, their combination appears as suggestive for efficient induction of apoptosis in melanoma.
