# Expression of Concern: General sensitization of melanoma cells for TRAIL-induced apoptosis by the potassium channel inhibitor TRAM-34 depends on release of SMAC

**DOI:** 10.1371/journal.pone.0323638

**Published:** 2025-04-28

**Authors:** The PLOS One Editors

After this article [1] was published, concerns were raised regarding results presented in Figs 4 and 6.

Specifically:

- Fig 4B A-375 GAPDH panel appears similar to Fig 4D A-375 GAPDH panel.
- The following panels appear similar:
  ◦ Fig 6D Bax (25), Bak (28) and GAPDH panels in [1], Fig 4e Bax, Bak, and GAPDH panels in [2], and Fig 5a Bax, Bak, and GAPDH panels in [3].
  ◦ Fig 6E Bcl-2 and GAPDH panels in [1] and Fig 4c Bcl-2 and GAPDH panels in [2].
- The following panels in [1] appear similar despite representing different experimental results:
  ◦ Fig 6C A-375 Bax and Fig 6D GAPDH
  ◦ Fig 6C A-375 Bcl-2 and Fig 6C A-375 Puma
  ◦ Fig 6C A-375 TS Bcl-2 and Fig 6C A-375 TS Puma

Regarding Fig 4B and Fig 4D GAPDH panels, the corresponding author stated that the Fig 4B A-375 DR5 and Fig 4D A-375 DR4 were run on the same blot, therefore the same GAPDH image was used. PLOS considers this concern resolved.

The corresponding author stated that the Fig 6D Bax, Bak, and GAPDH panels in [1] are the same as those in Fig 4e in [2] and Fig 5a in [3], and that the Fig 6E Bcl-2 and GAPDH panels in [1] are the same as those in Fig 4c in [2]. They also stated that these blots are for illustrative purposes only, and may not directly relate to the experiments conducted in [1].

The corresponding author stated that the Fig 6C A-375 Puma and A-375 TS Puma panels, and the Fig 6D GAPDH panel are incorrect, and the Fig 6C A-375 Bax and Bcl-2 panels, and the A-375 TS Bcl-2 panels are correct. They provided replacement images for the incorrect panels from replicate experiments conducted at the time of the original experiments (S1 File), but stated that the original data underlying the published and replacement panels are no longer available. In the absence of the original data underlying the published results, these concerns cannot be fully resolved.

The corresponding author stated that all underlying data for the results in [1] are no longer available.

The *PLOS One* Editors issue this Expression of Concern to alert readers to the above unresolved concerns in Figs 6C-D.

The Mel-HO cell line reported in [1] was identified as a misidentified cell line prior to the publication of [1], and is identical to the MEL-WEI cell line, a skin-derived melanoma line [4]. The corresponding author stated that they believe the MEL-HO cell line was correct, and that the MEL-HO cell line was purchased from Leibniz Institute DSMZ-German Collection of Microorganisms and Cell Cultures, Braunschweig, Germany. The authors did not provide STR profiling data to authenticate the cell lines used in this study. Readers are advised to consider the misidentified cell line concerns when interpreting reported results related to this cell line.

## Supporting information

S1 File Updated version of Fig 6. The updated panels for Fig 6C A-375 Puma, Fig 6C A-375 TS Puma, and Fig 6D GAPDH are from replicate experiments from the time of the original experiments.
(PPTX)
