# Supplementary figures and images for: Expression of Concern: General sensitization of melanoma cells for TRAIL-induced apoptosis by the potassium channel inhibitor TRAM-34 depends on release of SMAC
Source: PLoS One. 2025 Apr 28;20(4):e0323638. doi: 10.1371/journal.pone.0323638 (PMC12036924; doi:10.1371/journal.pone.0323638)

## Slide 1
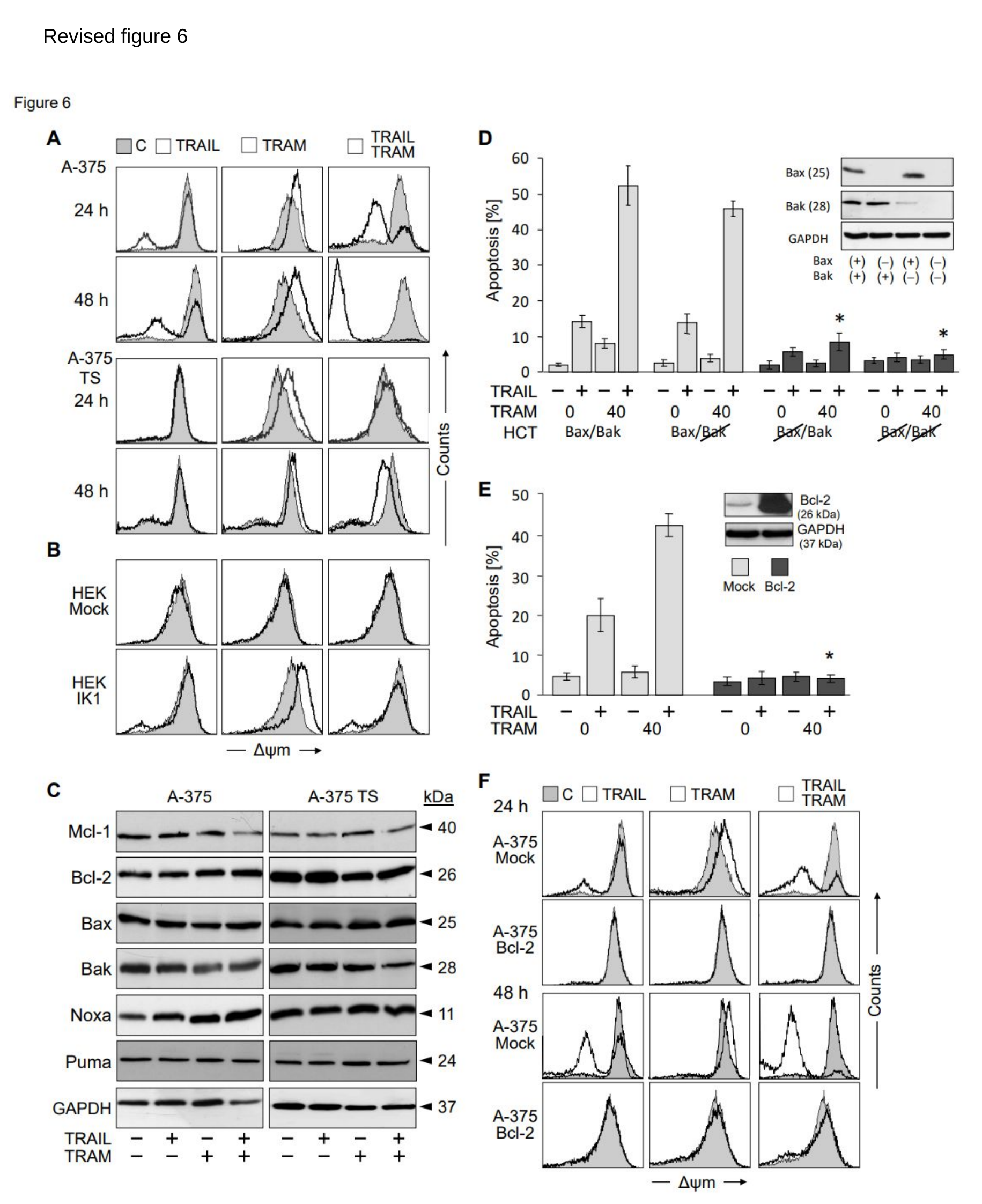

Revised figure 6
Δψm

Supplement: S1 File — The updated panels for Fig 6C A-375 Puma, Fig 6C A-375 TS Puma, and Fig 6D GAPDH are from replicate experiments from the time of the original experiments. (PPTX) [file pone.0323638.s001.pptx]
